# Platelet-inspired medicine for tumor therapy

**DOI:** 10.18632/oncotarget.22853

**Published:** 2017-12-01

**Authors:** Jie Yang, Shujun Wang, Ping Liu, Lu Dai, Baoan Chen, Jianfeng Luan, Jiaying Zhou

**Affiliations:** ^1^ Department of Hematology and Oncology, Zhongda Hospital, School of Medicine, Southeast University, Nanjing, People’s Republic of China; ^2^ Department of Blood Transfusion, Nanjing General Hospital of PLA, Nanjing, People’s Republic of China; ^3^ Department of Bioengineering, University of California, Berkeley, CA, United States of America; ^4^ Department of Radiology, Zhongda Hospital, School of Medicine, Southeast University, Nanjing, People’s Republic of China

**Keywords:** platelet, tumor, drug-loaded system

## Abstract

As the number of patients with tumor increases dramatically recent years, traditional therapies expose more and more problems which can even lead to death. Many researchers and clinicians quest for an efficient drug delivery system to deal with tumor as a result. With the researches further develop, we find that platelet can interact with tumor cells through a variety of ways. So it can be used as a carrier broadly to deliver different anti-tumor drugs for tumor treatment. In the present review, we summarize the interaction of tumor cells and platelet. At the same time, we focus on recent progress on the application of platelet drug-loaded system in the anti-tumor prospects.

## INTRODUCTION

Although traditional chemotherapy is still the main method in certain cancers, it has exposed much more serious problems and limitations in daily clinical work. Drug delivery system in a controlled manner is as important as discovery of new drugs [1].

The phase III PROCLAIM trial, recently published in the Journal of Clinical Oncology entitled “PROCLAIM: randomized phase III trial of pemetrexed-cisplatin or etoposide-cisplatin plus thoracic radiation therapy followed by consolidation chemotherapy in locally advanced nonsquamous non-small-cell lung cancer”, compared two different chemotherapy regimens given concurrently with radiotherapy in patients with stage III non-squamous lung cancer: pemetrexed plus cisplatin versus cisplatin plus etoposide [2]. This study was stopped as it showed no difference in effective rate and overall survival. Biomimetic drug delivery system offers new opportunities to mimic the biological particulates, including cells, vesicles and viruses for enhancing biocompatibility and promoting therapeutic efficacy [3].

Platelet is one of the visible components of mammalian blood. It is cytoplasmic bodies that are biologically active from cytoplasmic cleavage of megacaryocytes from bone marrow. It was treated as cell debris in the blood without function for a quite long time. With the study of platelet go into deep, we realize that platelets are much more complex than red blood cells (RBCs) and white blood cells (WBCs). While platelets are primarily confined to blood vessels, they may also accumulate in tumor microenvironments where they modulate functions of both tumor and immune cells [4]. Tumor cells can interact with platelets through a lot of receptors and signaling molecules. On this basis, more platelet drug-loaded systems are found in different basic and clinical researches which have reached pretty good results.

## PLATELET AND TUMOR’s INTERACTION

### Tumor-induced platelets activation

Multiple preclinical studies indicated that tumor cells and platelets maintain bidirectional interactions in the tumor microenvironment and enhance each other’s transformation via various mechanisms, involving direct content, alteration of gene expressions, release of cytokines and proteins, and shedding of microparticles, regulating each other’s functions. Tumor cells possess the ability to induce platelets aggregation, a behavior described as the tumor-cell induced platelet activation(TCIPA) [5]. TCIPA can happen through direct contact with tumor cells and mediators like ADP and proteinases. The previous experimental studies indicate that B-thromboglobulin and P-selectin are referred as the markers of platelets activation, increasing in the circulating blood of patients [6]. But some research data show effect of proliferative platelets is not mediated via platelet GPIba, GPIIbIIIa, or P-selectin in the ovarian cancer [7]. The glycoprotein Ib-IX-V-complex(GPIb-IX-V) and GPVI are mainly responsible for initial platelet activation, and interplays between GPIbα and (von Willerand factor(vWF) on collagen or the surface of activated platelets are vital for the following tethering and rolling of platelets [8]. Besides, the mechanism of TCIPA may as well be caused by the generation of matrix metalloproteinases (MMPs). Podoplanin expressed by some tumor cells, tissue factor and platelet factor 4(PF4) can be the platelet activation markers at the same time [9]. Although tumor cells could up-regulate platelets by releasing soluble factors, platelets could reciprocally change tumor cells’ behavior by platelet-derived microparticles, transferred to cell membranes of nearby tumor cells and increase matrix metalloproteinase production.

### Platelet-induced tumor immune surveillance

Platelet membrane is embedded with a great quantity of adhesion molecules, such as integrin, selectins and leucine-rich glycoproteins. After platelets activation, platelets and tumor cells bind with each other to form the platelet-tumor cell conjugates, which initially escaping immune surveillance, supporting tumor cells arrest and enhancing tumor adhesion on endothelium and invasive potentials of tumor cells. Karl Egan’s experiments *in vivo* demonstrate that the effect of platelets is most remarkable at arterial shear rates generated by blood flow [10]. Platelets could act as protective cloaks for tumor cells to cope with immune destruction [11].

### Platelet-induced extravasation and angiogenesis

When tumor cells plan to extravasation, they will first connect to the luminal side of endothelial cells (ECs), mediated by various adhesion receptors on the membrane of tumor cells and ECs [12]. After that, tumor can follow the trans-endothelial migration to realize the extravasation and colonization in the target tissues. During this process, growth factors released from specific granules and signaling pathways induced by contact of platelets with tumor can influence vascular integrity and the microenvironment of tumor-platelet aggregates [13]. In addition, platelets could also release ATP and ADP from genes granules to enhance the tumor cell extravasation [14, 15]. To summarize, increase in adhesion of tumor cells to ECs is notably important in the process of extravasation [16]. Platelets granules include different functional pro-angiogenic factors, contributing to the stabilization of newly formed vessels, like VEGF and PDGF [17]. The present study reveals that glycans can regulate angiogenesis via control the migration of endothelial cells and affecting EC survival and vascular permeability [18].

## INHIBITION PLATELET FUNCTION TO BLOCK TUMOR METASTASIS

Extensive evidence has shown that platelets support tumor metastatic progression by inducing epithelial-mesenchymal transition of cancer cells and by shielding circulating tumor cells from immune-mediated elimination. A variety of factors related to platelet can enhance the occurrence and development of tumors through different ways. For example, the study developed by Zhang et al showed that liposomal nanoparticles which bear the tumor-homing pentapeptide CREKA (Cys-Arg-Glu-Lys-Ala). This nanoparticles deliver a platelet inhibitor, ticagrelor, into tumor tissues to specifically inhibit tumor-associated platelets [19]. The nanoparticle could inhibit the tumor metastasis effectively *in vivo* and vitro through blocking the acquisition of an invasive phonetype that induced by platelet. Besides, many impact factors have been found during recent studies. The summary of several factors is shown in table 1.

**Table 1:** Summary of several factors

Factors	Characteristic	Principle
Platelet-Derived Growth Factor (PDGF)	A basic protein stored in platelet alpha granules that can promoting cell proliferation	Activating cell to transmitting signal into the cells which can induce cascaded waterfall effect including division of target cell proliferation
Platelet-Activated Factor (PAF)	A phospholipid secreted by activated innate immune cells that can induce platelet aggregation	(1) Triggering tumor growth by the G-protein coupled receptor (PAFR)(2) promoting angiogenesis and vascular permeability by activating VEGF expression
Platelet Factor4 (PF4)	a specific protein synthesized from platelet alpha granules that can neutralize heparin and promoting thrombosis	Inhibiting angiogenesis and growth and promoting apoptosis via STAT3 and phosphorylation of STAT3

### Platelet-derived growth factor (PDGF)

PDGF molecules are recognized as the key regulation in oncogenesis and angiogenesis, having four isoforms including PDGF-A-D, and two receptors including PDGFR-α and PDGFR-β. PDGF and its PDGFR can participate in carcinogenesis and tumor development. Activated PDGF can induce the phosphorylation of PDGF receptor and AKT and finally play an essential role in the proliferation [20]. *In vitro* studies indicate that PDGF-B could induce the tube formation capability of vascular smooth muscle cells (VSMCs), increase the migration of VSMCs and a reside in S or G2/M phase in the cell cycle. In this way, high expression of PDGF-B is related to the inhibition of tumor growth and proliferation [21].

### Platelet-activated factor (PAF)

PAF is a phospholipid, secreted by activated innate immune cells. It participates in different mechanisms in tumor development, such as the release of histamine in activated leukocytes. It has been found to trigger tumor growth by the G-protein coupled receptor (PAFR) and promote angiogenesis and vascular permeability by activating VEGF expression [22]. Through various techniques, researchers conclude that PAF could induce cell cycle arrest by reducing the expression of cyclin-B and increasing the expression of p21 and impair DNA damage via phosphorylated-ataxia telanglectasis and rad related in human mast cells [23]. In addition, the bidirectionally crosstalk between PAF and epidermal growth factor (EGF) suggest that PAF induced by EGF via cytosolic phospholipase A_2_ could cause the acts on the PAF-receptor to promote tumor progression in the ovarian cancer [24]. Reactive oxygen species (ROS) could activate ROS-p38-CK2-NF-κB pathway and PAF could promote tumor metastasis in the pulmonary cancer via this pathway [25]. In smoking patients, researches show that cigarette smoking may facilitate metastatic diseases via PAF accumulation and a subsequent increase in the motility of tumor cells [26]. Ravi P. Sahu at al find activation of PAF-R could exert an immunomodulatory affects which is relevant to neoplastic development [27]. In the colitis-associated cancer, PAFR antagonist Ginkgolide B could decrease colonic inflammation, tumor cell number and microvessel density, which in turn proves that positive effect of PAF in tumors [22]. Patrick C. Hackler and his colleagues demonstrate that PAF-R agonist can augment tumor tissue growth and lung metastasis in the murine Lewis Lung Carcinoma models, which indicate that PAF could modulate cancer progression in the lung [28].

### Platelet factor4 (PF4)

PF4 is a tetrameric chemokine released from alpha granules in the activated platelets. It has been reported to have an important role in hemostasis/thrombosis, the vascular wall and the immunogenic target.

In myeloma cells, PF4 is identified have a negative effect on the angiogenesis and growth and a positive effect on the apoptosis via downregulating signal transducers and activators of transcription(STAT3) and phosphorylation of STAT3 whichis associated with SOCS3 [29]. With immunohistochemistry, internalized PF4 together with endogenous PF4 in alpha granules could uptake by megakaryocytes to finally localize to alpha granules, which is mediated by lipoprotrein receptor related protein 1 (LRP1) partially [30].

## PLATELET DRUG-LOADED SYSTEM

Even though platelet has so tight relation with tumor and specifically target affinity for certain cells, the studies on platelet drug-loaded system are not very popular. Because platelet composition is much more complex than other cells in blood and it is unstable and hard to keep for a while. The Xu et al developed a novel drug delivery system for lymphoma treatment: DOX-loaded platelets that were conjugated with anti-CD22 monoclonal antibodies (mAbs) (DOX–platelet–CD22). Platelets are bio- and immune-compatible drug carriers that can prolong the circulation time of drugs. Anti-CD22 mAb-labeled platelets can precisely deliver DOX to tumor cells [31]. It was found that the two platelet membrane proteins, GPIIb-IIIa intergrins and P-selectins, on the surface of the liposomes containing the adriamycin-coated platelets, allowed the drug carrier to specifically recognize the metastatic activity of breast cancer cells in the blood [32]. Some scholars have found that chemically synthesized silica nanoparticles surface after activation of platelet membrane and tumor-specific apoptosis induced ligand kinase modification, but also to identify the blood circulation of metastatic breast cancer cells. It significantly reduce the breast incidence of lung metastasis [33]. In the study developed by Li and his colleague, a unique “microenvironment” was confirmed upon introduction of cancer cells of different types into circulation where activated platelets and fibrin were physically associated with blood-borne cancer cells. Inspired by the observation, they prepared the synthetic silica particles with activated platelet membrane along with surface conjugation of tumor-specific apoptosis-inducing ligand cytokine, TRAIL. And it decreased lung metastases in a mouse breast cancer metastasis model [34]. As the study developed by Dai author et al showed, they used platelets as vehicles for tumor-targeting with cytoplasmic-loading of kabiramide (KabC) that could inhibit platelets’ aggregation. Fluorescence microscopy studies found KabC-platelets that coupled with transferrin and Cy5 on surface, bind specifically to RPMI8226 and K562 cells [35]. Zhang’s work reported the polymeric nanoparticles enclosed in the plasma membrane of human platelets, which are a unique population of cellular fragments that adhere to a variety of disease-relevant substrates. They synthesized the platelet membrane-cloaked nanoparticles (PNPs) by fusing human platelet membrane with 100 nm poly(lactic-co-glycolic acid) (PLGA) nanoparticles. The cloaked nanoparticles have selective adhesion. In the animal experiment, docetaxel and vancomycin, respectively, showed enhanced therapeutic efficacy delivered by the platelet-mimetic nanoparticles. The nanoparticles provide a more effective approach to realize tumor-target treatment [36]. The research developed by Gu and his colleague showed that conjugating anti-PDL1 (engineered monoclonal antibodies against programmed-death ligand 1) to the surface of platelets can reduce tumor recurrence and metastasis after surgery. In this way, the platelet can be utilized as a carrier to load anti-PDL1 to target circulating tumor cells (CTCs) exactly [37].

## CONCLUSION

Tumor is still a changing problem in our clinical work and many studies attempt to explore different methods to deal with it. Recently, combining several immunotherapeutic trials on treating solid tumor have achieved successful results, but they still need more practice [38]. Although the researches of chemical synthesized nano-carriers are much more than platelet drug-loaded system, there are various problems in the literature report on the nano-carriers. Most liposomes can only carry hydrophilic drugs and are excreted quickly from the body and are susceptible to immune rejection [39]. Metallic nano-carriers are easy to stay in liver and kidney tissues and cause inflammation and toxicity.

The platelet membrane surface contains many kinds of proteins that have diverse functions, such as immune regulating function, function of adhesion and targeted for certain cell types have natural targeted affinity [40, 41]. So the drug-carriers modified by platelet membrane can significantly enhance the carrier targeting property. At the same time, in tumor’s formation, metastasis and other processes, platelets played an important role that can't be ignored. Tumor cells release a variety of cytokines to activate platelets on the surface receptors of platelet membranes. What’s more, activated platelets can release many biological molecules. These biological molecules can further activate nearby more platelets, react on tumor cells, and promote tumor cell proliferation, adhesion and metastasis. Platelet drug-loaded system in anti-tumor is not very popular, but it will be widely applied in tumor treatment based on its no rejection *in vivo*, high degradation rate, loaded more drugs, good targeting, longer half-time, flexible structure and so on.

Although platelets possess these advantages above, some deficiencies are found during the study. As the blood source is limited, the platelets are less. The obtained platelet-carriers are not easy to be kept for a long time as well. Then batch-to-batch variability of platelet membrane can't be ignored. With more superior platelet drug-loaded systems developed, they can benefit tumor patients in future.
